# An evaluation of oxygen systems for treatment of childhood pneumonia

**DOI:** 10.1186/1471-2458-11-S3-S28

**Published:** 2011-04-13

**Authors:** Alastair G Catto, Lina Zgaga, Evropi Theodoratou, Tanvir Huda, Harish Nair, Shams El Arifeen, Igor Rudan, Trevor Duke, Harry Campbell

**Affiliations:** 1 Centre for Population Health Sciences, College of Medicine and Veterinary Medicine, University of Edinburgh, UK; 2 International Centre for Diarrhoeal Disease Research, Bangladesh, Dhaka, Bangladesh; 3 Public Health Foundation of India, New Delhi, India; 4 Croatian Centre for Global Health, University of Split Medical School, Split, Croatia; 5 Centre for International Child Health, Melbourne University Department of Paediatrics, Royal Children's Hospital, Parkville, 3052, Victoria, Australia

## Abstract

**Background:**

Oxygen therapy is recommended for all of the 1.5 – 2.7 million young children who consult health services with hypoxemic pneumonia each year, and the many more with other serious conditions. However, oxygen supplies are intermittent throughout the developing world. Although oxygen is well established as a treatment for hypoxemic pneumonia, quantitative evidence for its effect is lacking. This review aims to assess the utility of oxygen systems as a method for reducing childhood mortality from pneumonia.

**Methods:**

Aiming to improve priority setting methods, The Child Health and Nutrition Research Initiative (CHNRI) has developed a common framework to score competing interventions into child health. That framework involves the assessment of 12 different criteria upon which interventions can be compared. This report follows the proposed framework, using a semi-systematic literature review and the results of a structured exercise gathering opinion from experts (leading basic scientists, international public health researchers, international policy makers and representatives of pharmaceutical companies), to assess and score each criterion as their “collective optimism” towards each, on a scale from 0 to 100%.

**Results:**

A rough estimate from an analysis of the literature suggests that global strengthening of oxygen systems could save lives of up to 122,000 children from pneumonia annually. Following 12 CHNRI criteria, the experts expressed very high levels of optimism (over 80%) for answerability, low development cost and low product cost; high levels of optimism (60-80%) for low implementation cost, likelihood of efficacy, deliverability, acceptance to end users and health workers; and moderate levels of optimism (40-60%) for impact on equity, affordability and sustainability. The median estimate of potential effectiveness of oxygen systems to reduce the overall childhood pneumonia mortality was ~20% (interquartile range: 10-35%, min. 0%, max. 50%). However, problems with oxygen systems in terms of affordability, sustainability and impact on equity are noted in both expert opinion scores and on review.

**Conclusion:**

Oxygen systems are likely to be an effective intervention in combating childhood mortality from pneumonia. However, a number of gaps in the evidence base exist that should be addressed to complete the investment case and research addressing these issues merit greater funding attention.

## Background

The fourth Millennium Development Goal laid out ambitious targets for reducing childhood mortality among children under five by two thirds, by 2015 [1]. Because it is the leading cause of child deaths in the world, combating pneumonia should be central to strategies for reducing childhood mortality [2-4].

Several pathophysiological mechanisms cause death from pneumonia, but sepsis and hypoxemia are the two key mechanisms. A recent review estimated the prevalence of hypoxemic pneumonia amongst young children presenting to health services each year at 1.5 to 2.7 million [5]. Although all of these children would benefit from treatment with supplemental oxygen [6], supplies are often unavailable and inappropriately utilised throughout the developing world [7-9]. It has been argued that introducing robust oxygen systems with well-trained and equipped staff could substantially reduce mortality from pneumonia [10].

To provide a systematic approach to priority setting in international health, The Child Health and Nutrition Research Initiative (CHNRI) has developed a common framework to score interventions that aim to reduce disease burden [11-15] and implemented this methodology for research prioritization in a wide range of contexts [16-20]. This paper aims to use this framework in order to assess oxygen systems as a method for reducing paediatric mortality from pneumonia and to enable the comparison of oxygen systems to other relevant interventions (see also other publications in this series).

## Methods

**Figure 1:**
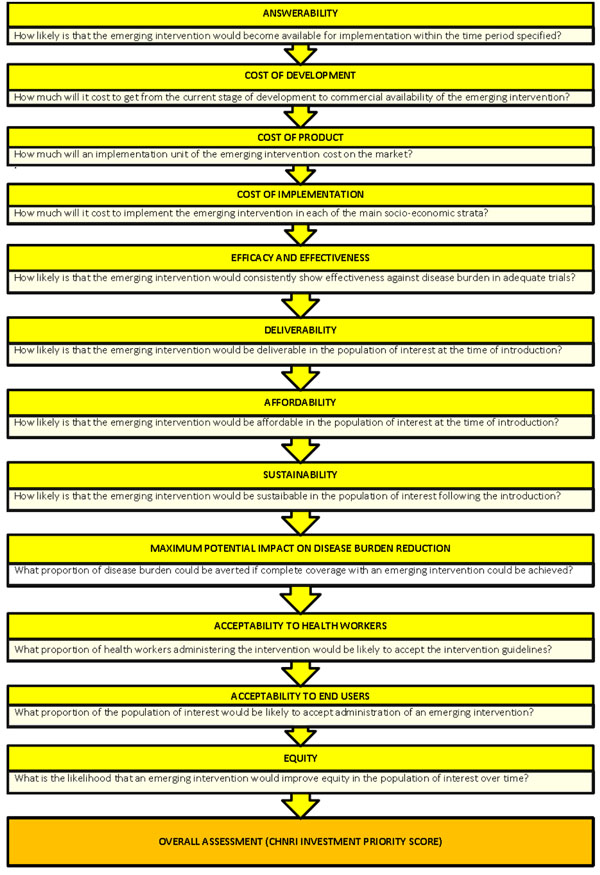
An illustration of the format of CHNRI approach for scoring interventions against childhood pneumonia.

For this project, a two stage CHNRI framework was used to assess the utility of oxygen systems. The first stage involved a thorough literature review of predefined criteria for scoring interventions against childhood pneumonia, laid out by CHNRI [11-15]. Criteria were chosen to best reflect the key elements of any intervention that should be taken into account for priority setting and included: (i) answerability, (ii) cost of development, (iii) cost of product, (iv) cost of implementation, (v) efficacy and effectiveness, (vi) deliverability, (vii) affordability, (viii) sustainability, (ix) maximum potential for disease burden reduction, (x) acceptability to health workers, (xi9 acceptability to end users, and (xii) effect on equity. An illustration of the format of this approach is outlined in Figure 1.

The second stage of the framework involved an expert opinion analysis, which made use of the opinions of 20 experts in relevant fields and included five basic scientists, five public health researchers, five international policy makers and five pharmaceutical representatives. Of these representatives, those involved in policy and pharmaceuticals participated on the condition of anonymity, because of highly sensitive nature of their involvement in similar exercises.

### CHNRI exercise – stage I: Identification and selection of studies

A literature search was performed for articles relating to oxygen therapy for paediatric hospital care in the developing world between 1950 and 2009. Using relevant keywords, we searched the databases of Pubmed, Pubmed Central, The Cochrane Library and those of developing countries including: LILACS - the Latin American and Caribbean Health Sciences Literature database, and IndMed - the Indian biomedical database. Titles and abstracts were reviewed for relevance. The references of relevant articles were screened to identify further useful articles.

A total of 315 articles were retrieved from PubMed, 862 from Pubmed Central, 22 from the Cochrane Library, 3 from IndMed and none from LILACS. After review of the titles of all articles and the references of those deemed useful, 96 full texts were located for inclusion in the study. Guidelines for oxygen use in developing countries were also located from the WHO website, and articles were taken from the International Union Against Tuberculosis and Lung Diseases. Details of the search and inclusion and exclusion criteria can be found in the Supplementary Table S1 in Additional File 1.

For sections covering Answerability and Efficacy, a separate search was conducted using relevant keywords, up to 2009**.** Historical accounts were useful, as only 8 journal articles were found to be of relevance. This is due to the early uptake of oxygen into medical practice discussed in the following sections. These historical accounts included internet resources and the WHO handbook (1993) [21].

### CHNRI exercise – stage II: An expert opinion exercise

To conduct the CHNRI expert opinion exercise, a preliminary review of the literature was provided and presented to the 20 experts that participated in a one week meeting held in Dubrovnik, Croatia, on September 7-13, 2009. The views of the experts were collected using a questionnaire designed to capture the opinion on all CHNRI criteria (Supplementary Table S2 in Additional File 1). The list of chosen experts included five leading basic scientists, five international public health researchers, five international policy makers and five representatives of the pharmaceutical companies.

The 20 experts were chosen based on their excellent track record in child health research, particularly childhood pneumonia. We initially offered participation to the 20 experts with the greatest impact of publications in their area of expertise over the past 5 years (for basic researchers and international public health researchers), or for being affiliated to the largest pharmaceutical company in terms of vaccination programme or international agency in terms of their annual budget. For those who declined to participate (4 experts - about 20% - mainly due to conflicting arrangements/travel), replacements were found using the same criteria: for basic scientists and public health researchers we used Web of Knowledge and “pneumonia or ALRI” as search subject and limited time period to 2001-2008. This gave us a larger number of papers, which we sorted according to number of citations received. Then, we went down the ranks and invited the corresponding authors of the studies that were most relevant to the topic of our expert panel. The policy makers and industry representatives accepted our invitation on the condition of anonymity, due to sensitive nature of their involvement in such exercises. About half of the experts were either affiliated to institutions in developing countries or had previous experience of working in developing country settings.

**Figure 2:**
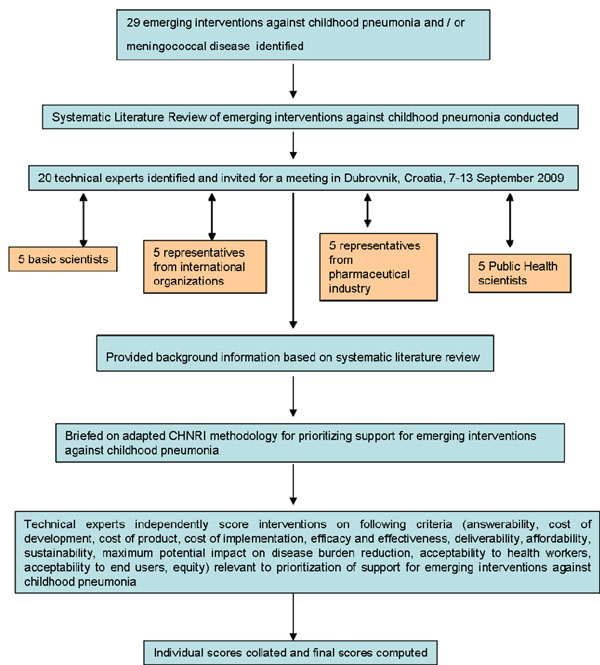
A summary of Stage II of the CHNRI process of an evaluation of emerging intervention (an expert opinion exercise using the CHNRI criteria)

The experts met during September 2009 to conduct the 2nd stage of CHNRI expert opinion exercise. All invited experts discussed the evidence provided in CHNRI stage I, and then answered questions from CHNRI framework Supplementary Table S2 in Additional File 1. Their answers could have been “Yes” (1 point), “No” (0 points), “Neither Yes nor No” (0.5 points) or “Don’t know” (blank). Their “collective optimism” towards each criterion was documented on a scale from 0 to 100%. The interpretation of this metric for each criterion is straightforward: it is calculated as the number of points that each evaluated type of emerging RSV vaccine received from 20 experts (based on their responses to questions from CHNRI framework), divided by the maximum possible number of points (if all answers from all experts are “Yes”). This was carried out for each question, dividing the sum of points received by the maximum number of points that could have been achieved. The exact computation methods have been explained at length and presented elsewhere [11-15]. An outline of the second stage of the CHNRI format can be viewed in Figure 2.

## Results

### Answerability

Oxygen is a well established treatment for hypoxemic pneumonia. It has been used in medical practice since the end of the 18^th^ century, but it was placed on a firm theoretical base for pneumonia by Haldane in the 1920s [22]. His description of oxygen and carbon dioxide transport in the lung almost immediately rendered oxygen as the standard treatment of “anoxemia” [22,23]. Due to the early and enthusiastic uptake of this treatment, empirical evidence for its benefit is virtually non existent. Any fully randomised controlled trial to establish the efficacy of oxygen therapy would require the withholding of oxygen from a control group, but once the theoretical basis for oxygen was understood, withholding oxygen in this way immediately became unethical.

### Efficacy - The impact of the oxygen systems under ideal conditions

No controlled trial has ever measured the therapeutic impact of oxygen in humans directly, but it is possible to estimate the effect in other ways. For example, mortality rates from studies prior to the introduction of oxygen therapy can be compared with those afterwards [21]. Due to the variability in severity between pneumonia outbreaks, one must have an adequate indicator of disease severity to be enable a meaningful comparison.

In some early studies, arterial oxygen saturations were recorded without the administration of oxygen therapy [24]. The outcomes of these patients can be compared with those from a similar time period in which oxygen saturations were taken and oxygen therapy provided [21]. When adjusted for illness severity, this comparison shows a mortality rate of 39% amongst those treated with oxygen and 74% in those without [21]. Although this suggests a significant impact, the patient numbers in those studies were far too small to provide statistical significance - in the study of Stadie (1919) there were only 34 patients in non-oxygen group [24]. Furthermore, these studies all took place in the pre-antibiotic era (hence the very high case fatality ratios) and so are of questionable relevance to the situation today [25-28].

Studies on guinea pigs infected with streptococci support the benefit of oxygen therapy: for animals kept in air mortality was 94%, while it was 49% for animals kept in 50% oxygen [21]. This empirical evidence is insufficient to make estimates of efficacy for oxygen therapy, but the current clinical consensus strongly, and subsequent studies support the efficacy of oxygen therapy. There is a strong theoretical and experiential basis, founded on decades of beneficial experiences of oxygen therapy in clinical practice, such that it is now a universally accepted standard of care in the management of hypoxaemia. The incorporation into most treatment algorithms of severe pneumonia in the world throughout the last century further establishes the benefit of oxygen therapy [6,23,29,30]. Therefore, it can be safely assumed that oxygen is an important part of treatment in severe pneumonia.

### Efficacy and effectiveness - The impact of oxygen systems in the population

The effectiveness of an oxygen system depends upon three stages in its delivery: the correct identification of patients requiring treatment; an effective method of administration; and adequate monitoring and eventual discontinuation of the therapy.

Current WHO guidelines advise oxygen therapy for every child with “very severe pneumonia” [6], which is diagnosed when following signs are observed: cough or difficult breathing, plus at least one of the following: (i) central cyanosis, (ii) inability to breastfeed or drink (or vomiting everything), (iii) convulsions, lethargy or unconsciousness, or (iv) severe respiratory distress.

Numerous studies have been conducted into the sensitivity and specificity of these clinical signs in the detection of hypoxemia [26,31-33] and wide variability has been found in the reported accuracies. For example, Dyke and colleagues found the sensitivity of chest in-drawing to be 98% and its specificity to be just 7%, whereas Gutierrez and colleagues found them to be 59% and 63% respectively [32,34]. These figures do not improve much when using multiple signs; although sensitivity can be increased, it is often at the expense of unacceptable specificity. Even the WHO treatment algorithms can inappropriately treat up to 50% of patients [31]. Indeed, a recent Cochrane review concluded that “*there is still no clinical sign, model or score system that accurately identifies hypoxemic children*”, a position that seems valid on analysis of the evidence base [35]. In the setting of such clinical uncertainty an adequate diagnostic investigation is needed. The pulse oximeter represents such a device.  However, very few hospitals in the developing world have access to oximetry, despite persistent calls for uptake [10,36-38].

Modes of oxygen delivery include nasal prongs, nasopharyngeal catheters, nasal catheters, facemasks and head boxes [21]. The choice of method should be made according to the situation, but in most hospitals and for most children nasal prongs are recommended [21]. Nasal prongs demonstrate a similar therapeutic effect and lower complication rates than other methods [39,40]. Face masks and head boxes have high oxygen requirements which outweigh their benefits in a low resource setting [21,35,39,40].

With these three stages in mind, the true effectiveness of an oxygen system can be adequately assessed only through studies in which pulse oximetry was introduced, an appropriate oxygen delivery system used and adequately trained staff has diagnosed, monitored and discontinued therapy. To date, the only study that has formally evaluated the effectiveness an oxygen system whilst covering these three considerations was undertaken by Duke and colleagues in Papua New Guinea [10]. The mortality rates of more than 11,000 children were compared from five hospitals before (2001-2004) and after (2005-2007) the implementation of such a system. They found that pneumonia mortality decreased by 35% (p<0.0001) after the introduction of an improved oxygen system. Although methodical issues can be raised over the comparison of children from different time periods, the confidence intervals supplied (48% - 22%) were still highly significant. In addition to this there are reports of observational studies describing large scale programmes which have introduced an oxygen system and have reported subsequent falls in pneumonia case fatality ratios [41]. However, more research is needed to further assess effectiveness in different contexts, and to evaluate the potential for such systems to reach a national scale in developing countries.

**Figure 3:**
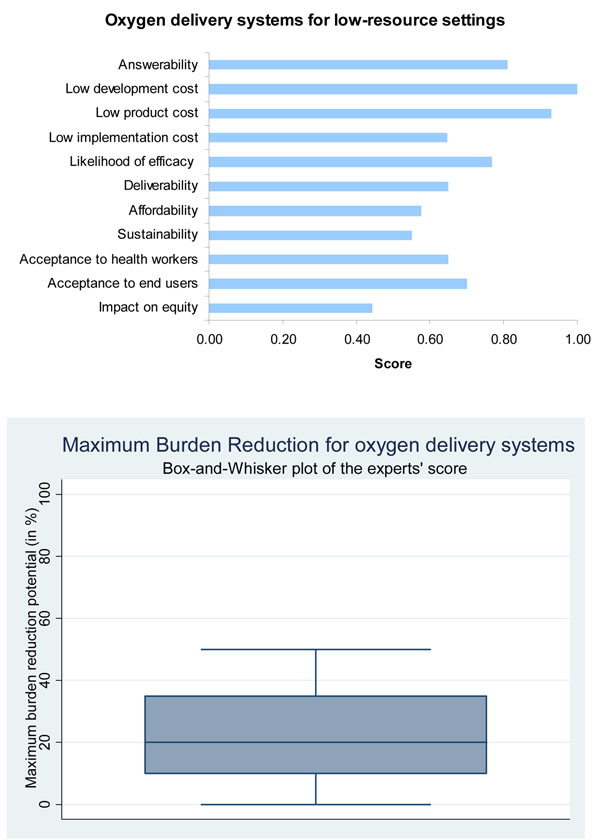
The results of Stage II CHNRI process – an expert opinion exercise assessing the potential usefulness of investment in oxygen. For Max Burden Reduction: Median (IQR): 20% (10, 35%), min: 0%, max: 50%

In this current exercise experts were highly optimistic (78%) about the effectiveness of oxygen therapy interventions for the reduction of deaths from childhood pneumonia Figure 3.

### Deliverability and sustainability

An accurate assessment of the current state of oxygen systems throughout the world would be invaluable for estimating the delivery requirements of improving oxygen capacity globally. Unfortunately, although research reports and accessible governmental data are becoming available on this topic there is still a need for further well designed studies to identify the major problems and suggest and evaluate appropriate strategies to tackle these. In some instances broader research questions, ranging from assessments of general hospital capacity to maternity ward facilities, include details about oxygen systems [7-9,42-44]. Although not all of these studies contain much detail on oxygen specifically, information can be extracted to aid understanding of the current capacity.

Table 1 shows how deficiencies differ greatly between regions. The countries of the former Soviet Union (e.g. Moldova, Kazakhstan and Russian Federation) have access to oxygen in between 72 and 95% of hospitals, but this oxygen supply is primarily available in Intensive Care Units rather than paediatric wards, and suffers from extensive equipment shortages. In several Russian hospitals oxygen was administered without flow meters and fed through shower heads to neonates [7]. In the Commonwealth of Independent States the primary requirements would be strengthening current systems with equipment and training [7].

**Table 1:** An availability of oxygen systems in different regions

Author	Date	Location	Health setting	Specific Availability	Number of facilities	Percent with oxygen	Supply outstrips demand
Kambarami	2000	Zimbabwe	Primary Health Care setting	Obstetric care	13	23%	n
Nolan	2001	Bangladesh, Dominican Republic, Ethiopia, Indonesia, Philippines, Tanzania, Uganda	Teaching and district hospitals	Paediatric care	21	77-87%	y
Simoes	2003	Uganda, Tanzania, Niger	Primary care facilities	Paediatric care	62	5%	y
English	2004	Kenya	Outpatient clinic	Paediatric care	14	14%	y
Wandi	2006	Papua New Guinea	Hospitals	Paediatric care	5	-	22% of children not treated
Duke	2006	Kazakhstan, Moldova, Russian Federation	Hospitals	Paediatric care	17	72-95%	y
Hill	2009	The Gambia	Health facilities	All areas	12	25%	y

On the other hand, more general deficiencies were found in the African region. A study in Kenya found that demand was outstripping supply in 11 out of 14 hospitals, and none had an oximeter [8]. A Tanzanian survey showed that 75% of hospitals had access to sufficient oxygen in less than 25% of the time [42]. These problems exist in some form or another throughout the developing world. A study of 21 hospitals in 7 developing countries ranging from Ethiopia to the Philippines showed that oxygen was only available in the emergency treatment areas of 47% of district hospitals [9]. Reports suggest that oxygen supplies are now reaching even the most remote parts of Africa, but sustainability is often prohibitive [45]. Overall, intermittent supplies, inadequate equipment and lack of training can be found in almost every region of the developing world [7,8,46,47].

In the past, a solution to this problem has been to accept foreign donations of oxygen equipment, but this has recently been demonstrated to be ineffective [48,49]. Without adequate training and maintenance, equipment can quickly fall into disrepair [50]. Using the evidence base it is possible to outline the equipment, infrastructure and funds that are necessary for implementing a system in most countries. It should be remembered that any planned upgrade of oxygen systems should be preceded by a review of a specific setting, current oxygen supply and attitudes of health workers and end users.

#### Equipment

There are two main sources of oxygen in hospitals: cylinders and concentrators. Oxygen cylinders contain the gas in compressed form. The empty cylinders have low capital costs (high cost when bought with flow-meters [51]), do not require an electrical supply but they need refilling at a supplier. The transport to the supplier can sometimes be challenging and costly in developing regions [45]. The gas itself is comparatively expensive at $4.80 to $6.56 per 1000 litres [51].

Oxygen concentrators are machines which can concentrate oxygen (up to 90%) at the bedside, by the absorption of nitrogen from atmospheric air. The product gas can be connected to up to four patients through flow splitters or a flow meter. Good quality concentrators delivering 5-10 L/min currently cost between $650 and $1500. They require an electricity supply, but produce oxygen at a far cheaper rate than cylinders. A study in Gambia estimated the cost per 1000 litres from concentrators at $0.84. This was calculated from a total cost in the period of 45 days when only concentrators were to be used in Medical Research Council Hospital, Fajara. The total cost comprised of estimated proportional concentrator capital (projected lifespan of 5 years) and running cost for this 45-day period, but it also included estimated proportional cylinder capital (projected lifespan of 20 years) and running costs in this period (because cylinders were used for 1 day, due to an oversight) [51]. They have sometimes been seen to pay for themselves within 6 months of purchase [52]. Concentrators have been tested in several regions of the developing world from Egypt and Malawi to Papua New Guinea with some success [10,48,52]. Those machines that fulfil both international standards (under the International Organisation for Standardisation) have been found most effective and durable when put to hard use [48].

There are several concentrator models on the market that are mostly appropriate for developing countries; however official WHO/UNICEF specifications have not yet been published [53,54].

For the reasons of cost and accessibility, many researchers have advised the use of concentrators [21,48,52]. However, although electricity is reaching ever more remote regions of the world, it can be unreliable [21,48,52]. A study in Malawi showed that cuts of greater than 3 hours were frequent in hospitals [48]. Howie (2009) developed an options assessment tool to determine the most appropriate form of oxygen supply in hospitals in Gambia [51]. Their analysis shows that if the national grid was constant, concentrators were far cheaper, showing an average annual facility cost of $18,742, rather than $152,747 for delivering oxygen using cylinders. However, electricity supplies were rarely available for 20 hours per day and the additional cost of stand-alone generators was not justifiable compared to cylinders. They developed a decision making algorithm based on their assessment tool, which stated that where an electrical supply was feasible or transport links unworkable, concentrators should be used; where electricity was unavailable but transport good, cylinders were appropriate. During their analysis they found that 10 out of 12 government facilities would in fact be more suited to cylinders than concentrators [51], but the authors noted that transport links to Gambian hospitals are generally good, which might not be true for other developing countries. In addition, other studies have shown transportation costs to reduce the sustainability of cylinders [45]. The trade off between concentrators and cylinders is complicated and decisions should be made according to setting. Advances in technology may eventually simplify this issue. Concentrators can be run off solar power [55], and those that fill cylinders directly are on the market [36]. However, this is currently very expensive: $25,000 for the solar panel to power a $650 concentrator (personal correspondence). Although these may not be appropriate in the developing world yet [51], they could eventually solve the problem of intermittent electricity. In addition, increasing access to electricity will favour the low cost oxygen of concentrators in future.

All equipment for oxygen systems should be bought with appropriate accessories and spares, such as nasal prongs, for the expected period of use [48]. This will increase the sustainability of the system as purchase of new spares from abroad can be problematic [48]. In addition, Matai (2008) has laid out guidance on selecting and purchasing oximeters for developing nations [56]. New oximeters are also in development, some with wind-up and solar power supplies specifically for use in remote hospitals [57].

The overall deliverability of oxygen received a score of only 0.64 in our expert opinion exercise reflecting the fact that the achievement of a successful oxygen delivery system nationwide is a complex and multidimensional challenge. A key element of this is to ensure proper ongoing maintenance of the system to maintain maximal function. This is why oxygen received a score of only 0.54 (out of maximum 1.00) on expert opinion for sustainability. Significant planning and infrastructure are required for successful implementation of oxygen systems.

#### Infrastructure

There have been several attempts to implement sustainable oxygen systems in developing regions, all using concentrators [48,52,56]. The experiences of these reports give valuable indications of the infrastructural requirements of an oxygen system. The most consistent observation is that prior to introduction, extensive training of staff is essential.

The day-to-day maintenance of concentrators is relatively easy, but capacity for regular servicing and repair must be available. Nursing and medical staff can be trained to clean the filters and perform general maintenance [21,56]. Minor malfunctions can often be repaired by local engineers while electro-medical engineers should be trained for major problems and visit every 4-6 months [21,48,52,56].

The comfort of health care workers in using such technology is an important factor in usage [48,58]. Medical personnel should be well versed in the indications, monitoring and discontinuation of oxygen therapy and with oximetry [56]. In a region with high staff turnover, training should be updated regularly to retain knowledge. This can be done by an annual visit from specialists at the central hospitals, or with the setting up of a national “oxygen team” [48,56].

### Cost of development and implementation and affordability

Two studies have outlined the costs involved in implementing a full, sustainable, oxygen system to paediatric wards; both used concentrators. In the first study by Enarson (2008), WHO approved concentrators were supplied to 22 district hospitals and 3 regional hospitals in Malawi [48]. In the second study in Papua New Guinea, the National Health department supplied 15 concentrators to 5 provincial and district hospitals [10]. Although costs will vary according to setting and time, a comparison of the expenditure of these two studies can be useful (Table 2).

**Table 2:** A comparison of two studies for the costs of oxygen systems.

Item	Enarson (2008)	Duke (2008)
Concentrators	$850	$2520
Installation materials	$1160	$830
Training and implementation	$1230	$970
Other (review visits and electro-medical repair)	$430	$2000
**TOTAL (per unit)**	**$3670**	**$6320**
Items relating to oximetry:		
Pulse oximeters and Oximetry sensor probes	Not available	$2280
**TOTAL (per unit and including pulse oximetry)**	**Not available**	**$8600**

Note: the cost of pulse oximeters and oximetry sensor probes was not estimated in the study of Enarson et al. (2008). The oxygen system is not complete without pulse oximetry

*All costs are averages for each unit rounded to the nearest 10.

These studies show that with adequate planning, training and repairs, oxygen concentrators can be sustainably implemented in district hospitals for an overall cost of between $3670 and $6320 per unit. The funds are split between equipment and human resources at roughly 55% vs. 45%, respectively. Both studies emphasise the benefits of investing in human resources. The actual cost of the concentrators was a low proportion of the total and over the first 5 years – in the low maintenance stage of a concentrators lifespan - becomes even less. In our own investigation, the average price of a suitable 4-litre concentrator was found to be between $650 and $1000.

If a cylinder system were chosen, in the best-case scenario the average annual cost to an average sized Gambian paediatric ward with the minimal leakage would be $5,000 per year [51]. This is compared to $1,500, in the best-case scenario for concentrator system with 24 hours of electricity per day and a five year life-span.

Pulse oximeters varied greatly in price [59]. Although very cheap models exist (<$100), those that have been used effectively in developing countries were generally more expensive [36,56]. The unit cost of an oximeter in the study of Duke and colleagues came to $2280. In addition, the disposable sensor probes were $217 each, and 30 were required to maintain seven oximeters for five years. Currently, suitable oximeters can be bought with a 5 year supply of sensor probes for $2000, roughly $10 per week of 5-year use [36]. A Global Pulse Oximetry Project has been launched which aims to encourage the development and distribution of suitable oximeters throughout the world (G.O. Project Report 2008). This project estimated the demand in developing countries to be over 100,000 units.

Cost effectiveness can be investigated by comparing patient outcomes with costs. Overall, in Papua New Guinea over 2.5 years in 5 hospitals 72 (CI: 52-94) children were estimated to have survived who would have otherwise died [10]. As the total cost of the project came to $120,462, the cost per additional life saved was $1673 (CI 1282-2317). If the Disability Adjusted Life Expectancy in Papua New Guinea is estimated at 33 then the price per DALY is $50 [10]. This is a conservative estimate. Many concentrators will last more than 5 years, the equipment will be used on children who have other hypoxemic conditions, and adjusted life expectancies are likely to rise in the future. The expert group score of 1.0 for development cost reflects the fact that this technology is already developed to a level that it is ready for implementation and no further major development costs are required (Figure 3).

### Maximum potential for disease burden reduction

Estimating the potential reductions in disease burden that oxygen systems could achieve is problematic given the current evidence base. An attempt can be made but the results should be treated with caution. A recent systematic review of 21 studies has estimated that between 1.5 and 2.7 million children consult health facilities with hypoxemic pneumonia worldwide each year [5]. According to WHO guidelines, every one of these children should receive oxygen therapy [21].

The death rate amongst hypoxemic pneumonia, as opposed to pneumonia without hypoxemia, can be estimated through analysis of studies measuring oxygen saturations. If the 35% reduction in mortality found in the Duke study could be replicated by improving oxygen systems in all regions, it could be expected to save between 68,000 and 122,000 lives. Because only one study was used to drive this global estimate, it should only be used as a rough guide and generalisation should be performed with caution before further evidence is gathered.

During our expert opinion analysis, the median maximum disease burden reduction of oxygen systems against childhood pneumonia was estimated at 20%, interquartile range of 10-35% (Figure 3).

### Acceptability and equity

Generally, oxygen is an acceptable intervention to both health workers and end users. However, it should be emphasised that the benefits of improving oxygen systems will only be felt by the 1.5 to 2.7 million children who consult the health services. The most vulnerable group in society, who are beyond the reach of the health sector, will not benefit. For this reason the intervention can be expected to reduce mortality from pneumonia but may not improve levels of inequity. On the other hand, it is also possible that through a strengthening of capacity in health services, the intervention might bring more of the population within reach of the health sector. Equally, falling case fatality rates and improved quality of care may result in higher levels of health services utilisation by all sectors of the population so the overall impact on equity is uncertain. There is no research on this subject and it is an area that would benefit from further investigation. The expert analysis demonstrated these doubts about the potential impact of oxygen systems in reducing child health inequities yielding a score of only 0.44, whereas the optimism over acceptability to both health workers and end users was high (60-80%) (Figure 3).

## Discussion

Although methodologically imperfect, this report provides valuable insights into the utility of oxygen systems and areas of uncertainty surrounding them. The limited efficacy data for supplemental oxygen in pneumonia is problematic, but addressed by decades of experience throughout the world. More robust evidence for effectiveness in different context and at a national scale is needed if investment in the area is to occur. The common consensus and theoretical understanding attempt to address this; however, multiple studies similar to those in Papua New Guinea [10] but covering other regions of the world would place oxygen systems on a far firmer evidence base. If the 35% reduction in mortality found in this study could be replicated in other regions, oxygen systems would appear much more likely to have a significant part in combating pneumonia. The highly conservative and rough estimates for mortality reduction of between 68,000 and 122,000 lives are encouragingly large.

Unlike other interventions such as antibiotics and vaccines, oxygen can be used to combat any form of hypoxemic pneumonia, regardless of its aetiology. It may be as effective at treating the various patterns of acute respiratory diseases in neonates, as well as other hypoxemic conditions found in children [5,60]. In addition, improved oxygen systems could have the added benefit of strengthening existing health facilities [10].

The deliverability of oxygen systems has never been more practical. Development of oxygen concentrators in the 1960s combined with recent research into their implementation in Malawi, Egypt and Papua New Guinea have greatly advanced the potential for building sustainable systems in regions with electricity. A systematic process for introducing equipment and training staff can now be outlined and implemented [10,48,52,56]. Finally, the cost effectiveness of $50 per DALY and cost per life saved of $1673 compares favourably with several other interventions against pneumonia [10]. For most countries however, accurate estimates of cost will be impossible until there is an improvement in the information on current oxygen capacities (Table 1).

There are a number of limitations in this review that deserve mention. Although the search strategy used was very broad, it may have been preferable to develop specific search terms for each criteria. This could have reduced the need for pro-active reference screening and unpublished report searching that was necessary in this review and detracted from its systematic nature. In addition, the absence of articles in foreign languages is problematic, as non-English speaking countries are so widely referred to. Future studies should include articles in other languages. Finally, attempts were made to update cost data for published reviews of concentrators [53]. This was done by contacting the manufacturers directly and would have been valuable in estimating the current costs of purchasing equipment. However, as only three of six manufactures replied, a general overview instead of a fully updated table was produced.

## Conclusions

An oxygen system could be a useful intervention in combating childhood mortality from pneumonia, with a possible potential disease burden reduction estimated at 20% (Figure 3). It is now feasible to implement and sustain an oxygen system, even in the most remote regions of the world. Cost of development is estimated to be low, although additional improvements of the existing systems are possible. However, a lack of evidence on effectiveness and sustainability in different contexts may hamper the investment potential. Further research on these issues would be highly valuable.

This review can be used to compare oxygen systems with other child health interventions, by referring to the other reports in this series and particularly to their expert analysis scores. This should inform decision making for investment decisions in future interventions to tackle pneumonia mortality in the future [1].

## Competing interests

The authors declare that they have no competing interests.

## Authors' contributions

AC performed the analysis, interpreted the data and wrote the manuscript. LZ, ET, TH and HN interpreted the data and wrote the manuscript. HC and IR designed the study and provided guidance in analysis and writing of the manuscript. TD and SEA revised the manuscript. All authors read and approved the final manuscript.

## Supplementary Material

Additional File 1 contains two supplementary tables: Supplementary Table S1: Details of the literature search and inclusion and exclusion criteria. Supplementary Table S2: Questions used in the Phase II CHNRI process Click here for file
